# Effect of retreatment on the push-out bond strength of MTA-based and epoxy resin-based endodontic sealers

**DOI:** 10.15171/joddd.2017.008

**Published:** 2017-03-15

**Authors:** Hamidreza Yavari, Shahriar Shahi, Saeedeh Galledar, Mohammad Samiei, Maryam Janani

**Affiliations:** ^1^Department of Endodontics, Faculty of Dentistry, Tabriz University of Medical Sciences, Tabriz, Iran; ^2^Dental and Periodontal Research Center, Tabriz University of Medical Sciences, Tabriz, Iran; ^3^Department of Endodontics, Faculty of Dentistry,Ardabil University of Medical Sciences, Ardabil, Iran

**Keywords:** MTA Fillapex, push-out bond strength, sealer, retreatment

## Abstract

***Background.*** Further studies on the adhesion properties of MTA-based materials seem necessary due to their growing use in endodontic treatment. This research aimed to assess the effect of retreatment on the bond strength of MTA-based (MTA Fillapex) and epoxy resin-based (AH Plus) sealers.

***Methods.*** ProTaper rotary files were applied to prepare the root canals of 80 human mandibular premolars. Then, the roots were randomly divided intotwo groups of A (n=40) and B (n=40), which were obturated with gutta-percha and MTA Filla-pex and AH Plus sealer, respectively. In both groups, the teeth were randomly subdivided into 2 subgroups. No retreatment was carried out in subgroups A_1_ and B_1_, while subgroups A_2_ and B_2_ were retreated with rotary files and a solvent. Then, a push-out test was performed on four 2-mm slices of each tooth at a distance of 2 mm from the coronal surface after two weeks of incubation. Data were analyzed with two-way ANOVA and statistical significance was set at P<0.05.

***Results.*** Regardless of the procedure followed (P<0.001), significant differences were detected in the mean bond strength values between the two sealers. Irrespective of the sealer type (P=0.3), no significant differences were revealed by comparing the mean bond strength values of the study subgroups. Furthermore, no statistically significant interaction (P=0.5) was found between the treatment and sealer types.

***Conclusion.*** AH Plus sealer exhibited a higher bond strength compared to MTA Fillapex. Retreatment using rotary files and chloroform had no statistically significant effect on the bond strength of sealers evaluated in this study.

## Introduction

Endodontic treatment may lead to the development of inflammatory periradicular lesions; therefore, it is not always successful. In most cases, the microorganisms survivingthrough the endodontic treatment procedures can penetrate into the root canal via coronal leakage, thus inducing treatment failures.^1,2^ Therefore, a non-surgical endodontic retreatment is required to restore health to the periapical tissues in such cases.^3^

Various techniques, including application by hand, as well as rotary and ultrasonic instruments, are employed to eliminate root canal filling materials. Softening of gutta-percha by heat or solvents can facilitate the mentioned steps.^4^The bond strength between root canal filling materials and root canal wall dentin may be influenced by the endodontic retreatment steps. Dentin surface chemical composition can be changed by root canal wall dentin exposure to some solvents, such as chloroform, during retreatment.^5^The strength of C&B Meta bond to root canal dentin was shown by Eldemir et al^6^ to be deleteriously affected by chloroform.

Biologic MTA-based sealers were developed since no endodontic sealers were available with ideal properties.^7^MTA Fillapex (Angelus, Londrina, PR, Brazil), composed of MTA, silica nanoparticles, silicate resin, natural resin, bismuth oxide and pigments, came to be applied as a new silicate-based sealer. Good physicochemical properties of this sealer have been found by recent studies, which include proper radiopacity and setting time, alkaline pH, high flow rate, and low solubility and absorption of water.^8,9^As shown in previous studies, AH Plus epoxy resin sealer has many advantages compared to other materials, providing a gold standard for evaluation of endodontic sealers in terms of resistance to dislodgment.^10,11^As compared to AH-plus sealer, MTA Fillapex sealer showed proper resistance to dislodgment in the root canal based on the push-out test results of a study conducted by Assman et al.^7^

Further studies are required to investigate the adhesive properties of MTA-based materials as they are ever-increasingly used in endodontic treatments. However, only a few studies have been so far aimed at the possibility of endodontic retreatment by MTA Fillapex sealers,^12^without an evaluation of their effects on the push-out bond strength using new techniques. Thus, the current study aimed to assess the effects of endodontic retreatment on the push-out bond strengths of MTA Fillapex and AH Plus sealers.

## Methods

A total of 80 single-rooted teeth of mandibular premolars with similar morphologies were selected. A standard root length of 14 mm was achieved by removing the toothcrowns. To ensure the root canal patency and determine the working length, a#10K-file(ManiInc,TochigiKen,Japan) was placed in each root canal. The working length was measured at 1 mm shorter than the file length as soon as its tip was visible at the apical foramen. Then, to standardize the apical foramen, a#20K-filewas applied. Using the crown-down technique, preparation of the root canals was carried outwith ProTaper rotary files (Dentsply, Maillefer, Ballaigues, Switzerland). In the cervical area, canal shaping was carried out with S1 and Sx files, followed by S2 file in the middle area and the finishing files F1, F2 and F3 in the apical area up to the working length. After each instrument, the root canals were irrigated with 5 mL of 2.5% NaOCl. After applying 10 mL of normal saline solution, 17% EDTA was used to flush the root canals for 1 min following thecompletion of cleaning and shaping steps. Paper points (Dentsply, Maillefer) were applied to dry the root canals, which were to be obturated. Based on the sealer type applied, 2 groups of 40 samples were randomly prepared:

**Group A:** MTA Fillapex (Angelus Industria de ProductosOdontologicosLtda, Londrina, Brazil)

**Group B:** AH Plus (De Trey, Dentsply, Konstanz, Germany)

Using a lateral compaction technique, the root canals were obturated with gutta-percha and sealer in both groups. Then, to help the sealers completely set, the teeth were incubated at 37°C and 100% relative humidity for 2 weeks. At this stage, 2 subgroups of 20 samples were randomly assigned out of each group. The samples of subgroups A_1_ and B_1_were prepared for the push-out test without a retreatment. However, using ProTaper Universal rotary retreatment files and chloroform as a solvent, the root canals of subgroups A_2_ and B_2_ underwent retreatment. Using a #2 Gates-Glidden drills (MANI Inc., Tochigi, Japan), 3 mm of the coronal parts of the root canal walls was removed. Then, each root canal receivedan injection of 3 drops of chloroform (Merck, Darmstadt, Germany). D1, D2 and D3 files were used for the evacuation of the coronal third and in the middle and apical thirds of the root canal up to the working length, respectively. F1, F2 and F3 finishing files were used for the final apical preparation. The similar irrigation and drying steps as those taken during the initial endodontic treatment were followed. Obturation of the root canals in the A_2_ and B_2_ subgroups was carried outusing gutta-percha sealerwith MTA Fillapex and AH Plus sealer via a lateral compaction technique, respectively. After 2 weeks of incubation at 37°C and 100% relative humidity, the samples were prepared for the push-out test.

### Push-out test 

A similar approach was taken to prepare the samples of all the subgroups for the push-out test. Using a diamond saw (SP 1600 Microtone, Leica, Nuβloch, Germany) under water spray, 4 dentin disks of 2 mm thickness were made perpendicular to the root long axis at a distance of 2 mm from the root coronal surface. To ensure the canal central positioning and homogeneous sealer layer with no bubbles,the coronal and apical surfaces of the disks were assessed. Since a plugger of 0.8 mm was used in this study, only samples with a canal diameter of 0.88±0.02 mm were selected for the push-out test after being measured with a digital caliper.

A digital caliper and special marker were used to measure the coronal and apical diameters and mark the apical area of the disk samples selected for the push-out test, respectively. Then, using a cylindrical stainless steel plugger of 0.8-mm diameter and a universal testing machine (Hounsfield test equipment, Model H5K-S, Surrey, England), a dislodging force was applied to the root canal filling material in an apico-coronal direction at a crosshead speed of 0.5 mm/min. The maximum force was converted to MPa after the dislodging force was measured in Newton by the following formula:

Push-out bond strength (MPa) = Maximum load (N)/Adhesion (mm^2^)

Calculation of the adhesion area was carried out through the following formula: π(R+r) [h^2^ +(R-r)2],^0.5^where π = 3.14 and R, r, and h indicate the coronal and apical radii and slice thickness, respectively.

To determine the effects of independent material variables (MTA Fillapex and AH Plus sealers), treatment type (retreatment or no retreatment), and the interaction between themon the push-out bond strength, two-way ANOVA was used to analyze the data. Statistical significance was set at P<0.05.

## Results

**Figure 1.**
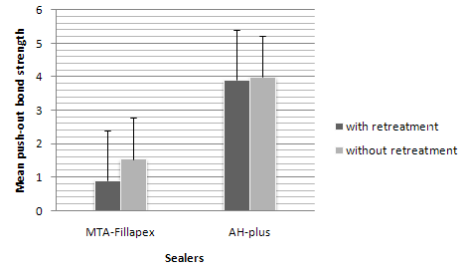
Graphic representation of the sealer`s mean push-out bond strength with or without retreatment.

The mean values of the push-out bond strength for the study groups are shown in Table 1. Regardless of the treatment type (P<0.001), a significant difference was found between MTA Fillapex and AH Plus sealers based on the mean bond strength values obtained via two-way ANOVA. Compared to MTA Fillapex sealer, AH Plus sealer represented a higher bond strength. However, no significant differences were observed between the bond strength means relevant to the treatment type regardless of the sealer type (P=0.3). Moreover, no significant interaction was seen between the treatment and sealer types (P=0.5; Figure 1).

**Table 1:** Push-out bond strength values (means ± standard deviations) for different groups

**Group**	**Type of treatment**		**Total**
	**Without retreatment**	**With retreatment**	
**Group A: MTA Fillapex**	1.54(±1.1)	0.9 (±1)	1.2 (±1.1)
**Group B: AHPlus**	4(±2.5)	3.9(±1.3)	4(±2)

## Discussion

The bond strength between dentin and the materials plays an important rolein the success of endodontic procedures.^13,14^To prevent sealer dislodgement under the mechanical stresses caused by operative procedures, tooth flexure, and post space preparation, sealer‒dentin adhesion is necessary under dynamic conditions.^15-17^ In this research, higher push-out bond strength was achieved with AH Plus compared to MTA Fillapex sealer. Nevertheless, higher push-out bond strength with AH Plus epoxy resin sealer has been reported by some previous studies.^18-21^The higher values of bond strength associated with epoxy resin sealers have been ascribed to the covalent bond between epoxide (open circle) and the exposed amino groups in collagen, low polymerization stress, and long-term dimensional stability.^19,21,22^

The bonding behaviors of MTA-based sealers can be affected by their chemical compositions. Resistance of MTA against dislodgement is promoted by its biomineralization capacity. However,due to the presence of resin components in MTA Fillapex sealer structure and decreased adhesion of apatite tag-like structures, a reduced bond strength to dentin willbe achieved.^8,12,22^ Their sealing and adhesion properties might be influenced by their solubility as well.^23^According to the results obtained by Borges et al,^24^ the bond strength differences of MTA Fillapex and AH Plus sealers can be attributed to the much higher solubility of MTA Fillapex sealer.

Based on the results of the present research, no significant effects on the push-out bond strengths of MTA Fillapex and AH Plus sealers are exerted by retreatment with rotary files and a solvent. As shown by a large number of previous studies, the time required for retreatment is reduced by a solvent^25-28^ since it can facilitate the penetration of rotary files into gutta-percha by plasticizing it andas evidenced by Carpenter et al,^29^application of chloroform as a solvent with MTA Fillapex retreatment would lead to the re-achievement of apical patency in 100% of cases and thus, it was used as the solvent in the present study. Nonetheless, some studies have shown the detrimental effects of using a solvent on the bond strength of root canal filling materials.^30,31^ In this regard, the negative effects of solvents on the bond strengths of AH Plus and Epiphany resin sealers were demonstrated in a study byNasim et al.^30^ Also, a significantly reduced bond strength of the filling material was discovered, resulting from the use of chloroform for Resilon/Epiphany SE retreatment by Shokouhinejad et al.^31^ These differences might be explained by differences in methodologies, the amount ofsolvent used and the duration of contact between the solvent and dentin in the present and previous studies. For instance, Shokouhinejad et al performed chemical flushing with a higher amount of chloroform and wicking procedure on each tooth 3 times. In this investigation, only one solvent, i.e. chloroform, was assessed for its effects on the bond strengths of the sealers during retreatment and thus, further research should be carried outon the effects of various solvents with different volumes on the bond strengths of various sealers under diverse retreatment techniques.

## Conclusion

AHPlus sealer exhibited a higher bond strength compared to MTA Fillapex. Retreatment using rotary files and chloroform had no statistically significant effect on the bond strength of sealers evaluated in this study.

## Acknowledgments

The authors would like to appreciate the Dental and Periodontal Research Center, Tabriz University of Medical Sciences, for the financial and technical support of this research project.

## Authors’ contributions

HY and SG contributed to the concept and design, SS and SG critically revised the manuscript.MS and SS contributed to data acquisition and interpretation, and drafted the manuscript. SS and MJ performed data analysis. All the authors have read and approved the final manuscript.

## Funding

This research was carried out by financial support of the Dental and Periodontal Research Center, Tabriz University of Medical Sciences.

## Competing interests

The authors declare no competing interests with regards to the authorship and/or publication of this article.

## Ethics approval

This research was approved by Research Ethics Committee of Tabriz University of Medical Sciences in 2014.
